# Elucidating the mechanistic link: how silicon enhances lodging resistance in oat via targeted regulation of lignin biosynthesis in the second stem internode

**DOI:** 10.3389/fpls.2026.1787541

**Published:** 2026-03-02

**Authors:** Lin Yang, Kexin Liu, Junying Wu, Fengwu Wang, Chengzhong Zheng, Qianjun Wang, Sairu Li, Xiquan Wang, Baoping Zhao

**Affiliations:** 1 College of Agriculture, Inner Mongolia Agricultural University, Hohhot, Inner Mongolia, China; 2 College of Vocational and Technical, Inner Mongolia Agricultural University, Baotou, Inner Mongolia, China; 3 Ulanqab Academy of Agricultural and Forestry Sciences, Ulanqab, Inner Mongolia, China

**Keywords:** lignin, lignin-biosynthetic enzymes, lodging resistance, mineral elements, oats, silicon input

## Abstract

**Introduction:**

Oat production is constrained by lodging, and silicon input has been shown to promote lignin accumulation in basal internodes and enhance stem mechanical strength and lodging resistance. However, the physiological mechanisms by which silicon input regulates lignin biosynthesis in the second basal internode of oat stems and its effects on lodging-related traits remain unclear.

**Method:**

A split-plot field experiment was conducted in 2024 and 2025, with Mengyan 1 (MY1, lodging-resistant) and Dingyan 2 (DY2, lodging-susceptible) assigned to the main plots and five silicon inputs (0, 30, 60, 90, and 120 kg ha^-1^) to the subplots. Lodging-related and physiological traits were analyzed at the grain-filling and milk stages, and the dynamic patterns of lignin-biosynthetic enzyme activities were investigated.

**Results:**

MY1 exhibited the highest lodging resistance at a silicon input of 60 kg ha^-1^, and its lignin content increased by 12.5% and 14.6% at the grain-filling and milk stages, respectively, compared to no silicon input. In contrast, DY2 achieved the strongest lodging resistance at an input of 90 kg ha^-1^, with lignin content increasing by 12.4% and 17.0% at the two stages, on average in two years. Notably, stem lodging resistance was closely associated with lignin content of the second basal internode in grain-filling (*R*^2^ = 0.80) and milk (*R*^2^ = 0.64) stages. Silicon primarily enhances stem lodging resistance in oat by promoting lignin accumulation. This effect is achieved through the stimulation of lignin-biosynthetic enzyme activities and the accumulation of key mineral elements in the second basal internode, thereby markedly increasing stem lignin content. Random forest analysis indicated that cinnamyl alcohol dehydrogenase activity at 30 days after jointing made the greatest contribution to lignin biosynthesis, whereas magnesium content at the grain-filling stage was the most influential mineral factor.

**Conclusions:**

Silicon inputs of 60 and 90 kg ha^-1^ are recommended for lodging resistant and susceptible oat cultivars respectively, and it enhances lodging resistance by the promotion of lignin accumulation through upregulating enzyme activities and increasing mineral content in the stems.

## 1 Introduction

Oat (*Avena sativa* L.) is an important dual-purpose cereal-forage crop (Liu et al., 2025). It is widely cultivated worldwide owing to its broad adaptability, rapid growth, and high contents of functional proteins, β-glucans, and other bioactive constituents (Patra et al., 2023; Fu et al., 2020). In recent years, with the advancement of agricultural mechanization and the adoption of high-density planting systems, changes in oat canopy structure have exacerbated lodging, which has become a major constraint on yield stability and industrial development of oat production (Liu et al., 2024a). Therefore, identifying lodging-resistant cultivation strategies adapted to high-density planting conditions is of substantial practical importance.

Lodging is a major constraint on achieving high and stable oat yields and is governed by multiple interacting factors, including environmental conditions, agronomic practices, and cultivar genetic characteristics (Mohammadi et al., 2020; Niu et al., 2022). According to the site of occurrence, lodging can be classified into root lodging and stem lodging, within which stem lodging caused by basal stem breakage is the most prevalent (Li et al., 2025; Yu et al., 2025). Notably, the earlier lodging occurs during crop development, the greater the resulting yield losses (Shah et al., 2017). Evidence indicates that lodging in oat mainly occurs within the first to third basal internodes (counting from the soil surface), and that the second basal internode serves as a critical load-bearing structure and is particularly susceptible to fracture (Wang et al., 2025; Zhang et al., 2016). The morphological traits and physiological characteristics of the second basal internode show a significant positive correlation with the lodging resistance index, and the mechanical strength of the second basal internode is considered a key indicator of stem lodging resistance (Yu et al., 2025). Physiologically, stem mechanical strength is closely associated with cell wall architecture, as lignin deposition in the secondary cell wall enhances stem rigidity and bending resistance, thereby improving lodging resistance (Li et al., 2021; Luo et al., 2019). In gramineous crops, stem lignin biosynthesis primarily depends on the phenylpropanoid pathway, with key enzymes including phenylalanine ammonia-lyase, tyrosine ammonia-lyase, 4-coumarate: CoA ligase, and cinnamyl alcohol dehydrogenase (Barros et al., 2016a). Meanwhile, lodging-resistant cultivars exhibit significantly higher concentrations of K, Ca, Mg, and Si in basal stems than lodging-susceptible cultivars (Yu et al., 2025). In addition, the activities of lignin-biosynthetic enzymes are closely associated with stem mineral contents, and their coordinated effects promote lignin biosynthesis and deposition, thereby enhancing stem mechanical strength and lodging resistance (Wu et al., 2023; Liu et al., 2018a).

Silicon an essential nutrient following nitrogen, phosphorus and potassium, is abundant in gramineous crops. however, long-term cultivation often results in insufficient plant-available Silicon in soils, which is inadequate to meet the requirements for normal crop growth and stress resistance (Wang et al., 2021; Manivannan et al., 2023; Kovács et al., 2022). After uptake by plants, Silicon is deposited as silica in epidermal cells and cell walls, forming silicified structures that increase cell-wall compactness and promote lignification, thereby enhancing lodging resistance (Rudall et al., 2025; Wu et al., 2025). Studies in major staple crops such as rice, wheat, and rapeseed have demonstrated that silicon input promotes mineral nutrient uptake, enhances the activities of lignin-biosynthetic enzymes and lignin accumulation, and simultaneously shortens basal internodes while increasing stem diameter and tissue compactness, thereby strengthening stem mechanical properties and ultimately improving stem lodging resistance (Dorairaj et al., 2017; Yang et al., 2024; Hu et al., 2022).

To date, most studies have focused on the effects of silicon on crop morphology or overall lodging resistance, whereas the physiological mechanisms by which silicon regulates lignin biosynthesis in the second basal internode, a critical site for lodging resistance, and the resulting impacts on oat lodging remain unclear. In addition, for oat cultivars with different lodging resistance, the optimal silicon input rate remains unclear. To address these gaps, a two-year field experiment was conducted using oat cultivars with contrasting lodging resistance to systematically evaluate the effects of graded silicon inputs on stem mechanical strength, lignin accumulation, lignin-biosynthetic enzyme activities, and mineral composition in the second basal internode, as well as to elucidate the interrelationships among these traits. The study aims to elucidate how silicon-mediated regulation of lignin biosynthesis in the second basal internode relates to lodging resistance, thereby providing a theoretical basis for silicon-based cultivation strategies to mitigate oat lodging.

## 2 Materials and methods

### 2.1 Experimental site

**Figure 1:**
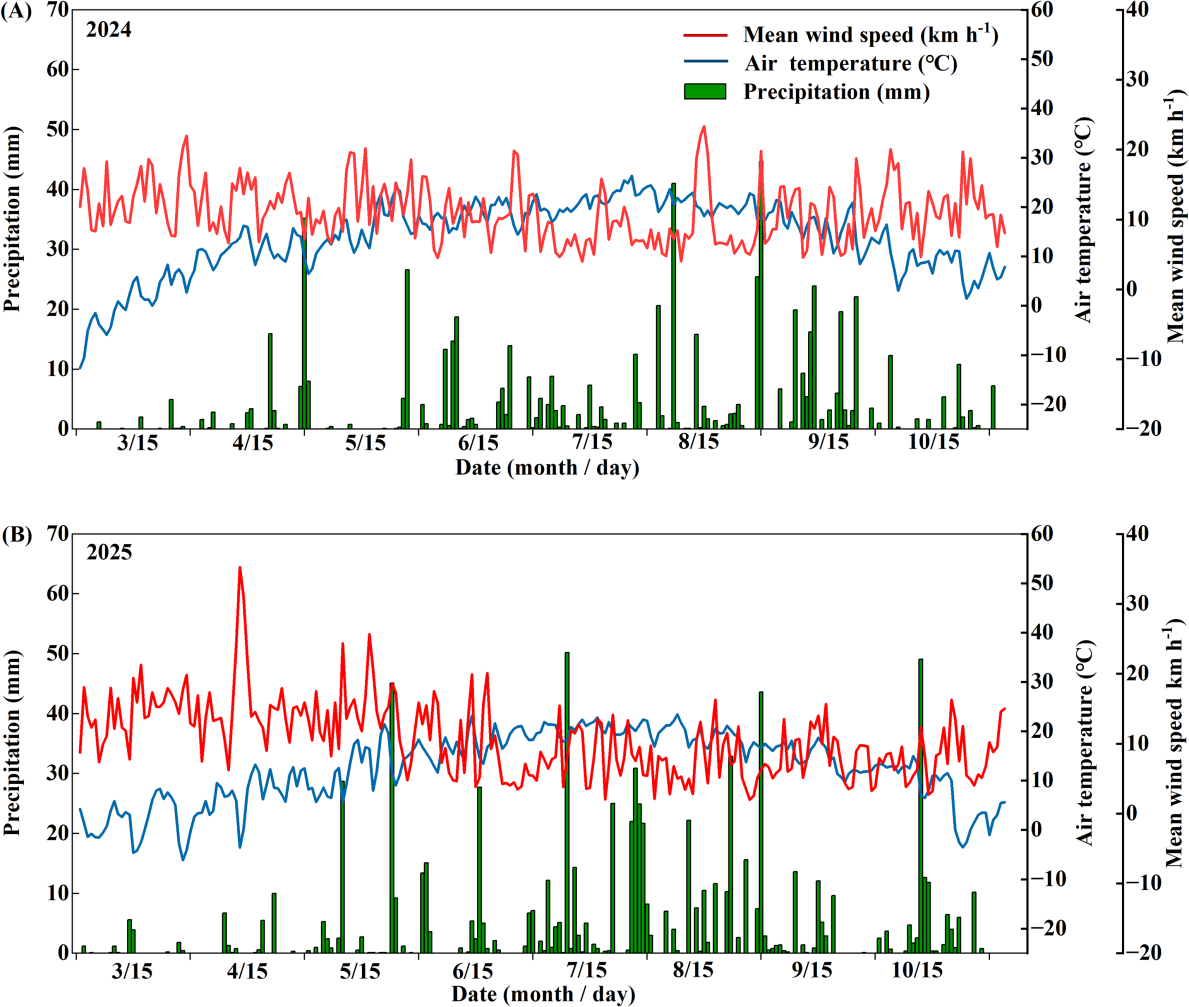
Daily mean precipitation (green bars), mean air temperature (blue line), and mean wind speed (red line) during the oat growing seasons in the experimental site from 2024 **(A)** to 2025 **(B)**.

The experimental site was located in Chahar Right Front Banner, Ulanqab City, Inner Mongolia Autonomous Region, China (40.92° N, 113.11° E). The region has a mean annual temperature of 9°C, an average frost-free period of approximately 120 days, and a mean annual precipitation of 360.8 mm. Precipitation, air temperature, and mean wind speed during the 2024 and 2025 growing seasons are shown in **Figure 1**. The soil is predominantly Calcisol, and the baseline soil fertility of the 0–20 cm layer is presented in **Table 1**. Field experiments were conducted in two consecutive years at different fields within the same area.

**Table 1:** Basic physical and chemical properties of soil in the experimental site from 2024 to 2025.

Year	Soil organic carbon (g kg^-1^)	Total nitrogen (g kg^-1^)	Total phosphorus (g kg^-1^)	Total potassium (g kg^-1^)	Alkaline- hydrolyzable nitrogen (mg kg^-1^)	Available phosphorus (mg kg^-1^)	Available potassium (mg kg^-1^)	Available silicon (mg kg^-1^)	pH
2024	14.20	1.92	1.08	17.55	74.32	27.83	159.85	168.48	8.37
2025	16.60	2.00	1.11	15.26	99.69	44.89	211.38	71.55	7.61

### 2.2 Experiment design and materials

A split-plot design was employed, with oat cultivar assigned to the main plots and silicon input assigned to the sub-plots. Two cultivars with contrasting lodging resistance were selected: the lodging-resistant Mengyan 1 (MY1) and the lodging-susceptible Dingyan 2 (DY2). MY1 was developed by the Inner Mongolia Academy of Agricultural and Animal Husbandry Sciences. It has a plant height of 105.5 cm, a panicle length of 16.3 cm, and a spreading panicle type. The number of grains per panicle averages 54.6, with a thousand-grain weight of 34.0 g. Its growth period is about 90 days, classifying it as a mid-maturing cultivar.DY2 was bred by the Dingxi Academy of Agricultural Sciences. It has a plant height of 128 cm, a panicle length of 24.0 cm, and a compact lateral panicle type. The grains per panicle average 83.7, with a thousand-grain weight of 27.1 g. Its growth period is approximately 115 days, making it a mid-to late-maturing cultivar. Sodium metasilicate (Na_2_SiO_3_, analytical grade ≥ 99.0%) was used as the silicon source at five input rates: 0, 30, 60, 90, and 120 kg ha^-1^, each with four replicates. Sodium metasilicate was uniformly mixed with the compound fertilizer and applied as a basal application to the respective treatment plots. The compound fertilizer was applied at a rate of 150 kg ha^-1^ (N: P_2_O_5_: K_2_O = 18: 18: 18). Additionally, urea was top-dressed at 75 kg ha^-1^ (Total N ≥ 46.0%) both before and after the jointing stage. Sowing was carried out on 15 May 2024 and 11 May 2025. The seeding rate was 180 kg ha^-1^. Each plot measured 20 m^2^ with a row spacing of 25 cm. Furrows were opened mechanically, followed by manual seed broadcasting and soil covering. Throughout the growing season, weeding and irrigation were performed according to standard local agronomic practices.

### 2.3 Determination of stem lodging resistance

During the grain-filling and milk stages, a 50 cm representative stem segment was collected from each plot, avoiding border and adjacent rows. From these segments, 15 uniformly growing, non-lodged plants were selected for agronomic trait measurements, which included:

Second basal internode length (cm): The length of the stem between the second node from the base and the third node was accurately measured using a ruler (counting from the soil surface).

Panicle fresh weight (g): The entire panicle was excised at the base, and fresh weight was determined using an electronic balance.

Stem breaking strength (N): Measured using a YYD-1A portable plant stem strength meter (Zhejiang Top Cloud Instrument Co., Ltd.). Leaf sheaths were first removed, and the second basal internode was placed in the instrument’s groove with a support span of 5 cm. The flat compression probe was positioned at the midpoint of the second basal internode, and force was applied downward at a uniform speed until the stem fractured. The recorded reading represents the stem breaking strength. The lodging resistance index was calculated using the following formula (Wang et al., 2019):


$$\text{Lodging resistance index }=\;\frac{\text{Stem breaking strength (N) }}{\text{Stem length (cm) }\times \text{ Fresh panicle weight (g)}}$$

### 2.4 Measurement of mineral elements, lignin content, and enzymatic activities

During the grain-filling and milk stages, a 50 cm representative stem segment was selected from each plot, avoiding border and adjacent rows. The second basal internode was excised, leaf sheaths were removed, and samples were placed in an oven at 105°C for 30 min to inactivate enzymes, then dried at 80°C to constant weight. The dried samples were ground for determination of mineral and lignin content.

Lignin content: Acid-insoluble lignin (Klason lignin) was measured using the sulfuric acid method (Schwanninger and Hinterstoisser, 2002). Approximately 0.5 g of dried sample powder was accurately weighed into an acid-resistant container, and 15 mL of 72% sulfuric acid was added. The mixture was hydrolyzed in a 30°C water bath for 60 min. About 7.5 mL of distilled water was then added to dilute the acid to 4%, followed by autoclaving at 121°C for 60 min. After cooling to room temperature, the hydrolysate was filtered through a pre-weighed glass fiber filter. The insoluble residue was repeatedly washed with hot distilled water until the filtrate was neutral, then the filter and residue were dried at 105°C to constant weight. The dried residue was ashed in a muffle furnace at 600°C for 4h to remove ash, and the ash mass was recorded. Lignin content was calculated as:


$$\text{Lignin content }(\%)\;=\;\frac{{\text{m}}_{1}-{\text{m}}_{2}}{{\text{m}}_{0}}\times 100\%$$

Where m_0_ is the sample weight (g), m_1_ is the weight of acid-insoluble residue (g), and m_2_ is the weight of the residue ash (g).

Lignin-biosynthetic enzymes: Sampling was initiated at 10 days after jointing in both cultivars and repeated at 10-day intervals for a total of five samplings. In each plot, a representative 50-cm stem segment was selected while avoiding border and near-border rows. The second basal internode was excised, leaf sheaths were removed, and samples were immediately stored in a dry-ice freezer. The activities of lignin-biosynthetic enzymes, including tyrosine ammonia-lyase (TAL), phenylalanine ammonia-lyase (PAL), 4-coumarate: CoA ligase (4CL), and cinnamyl alcohol dehydrogenase (CAD), were measured using ELISA kits.

Mineral content: Approximately 0.2 g of oat stem powder was weighed into a digestion tube, to which 5 mL of HNO_3_ and 4 mL of H_2_O_2_ were added. Samples were digested for 30 min in a microwave digestion system (Mars6-Xpress, CEM Technology Co., Ltd., Matthews, NC, USA). The digestion tubes were then placed on a temperature-controlled apparatus (XMTG-7000, Gongsheng Instrument Co., Ltd., Yuyao, China) at 120°C until no yellow fumes were observed. The digested solution was transferred to a 50 mL volumetric flask and brought to volume with ultrapure water. Potassium, calcium, magnesium, and silicon concentrations were determined using an inductively coupled plasma optical emission spectrometer (ICP-OES, Agilent 5800, Agilent Technologies Inc., Santa Clara, CA, USA) (Barros et al., 2016b).

### 2.5 Statistical analysis

Data were compiled in Microsoft Excel 2019 prior to statistical analysis. Analysis of variance (ANOVA) together with Fisher’s Least Significant Difference (LSD) test (*p* < 0.05) was used to identify the effects of treatments on all the analyzed parameters. Prior to ANOVA, data were tested for normality using the Shapiro-Wilk test and for homogeneity of variance using Levene’s test (IBM SPSS Statistics, version 25.0). The random forest model was constructed using the “randomForest” package in R (v4.3.3) under the R Studio environment (v4.2.3). Variable importance and its statistical significance were assessed via permutation tests implemented in the rfPermute package, while the overall significance of the model was evaluated using the A3 package. Graphs were created using Origin 2024.

## 3 Results

### 3.1 Lodging resistance index and mechanical strength

**Figure 2:**
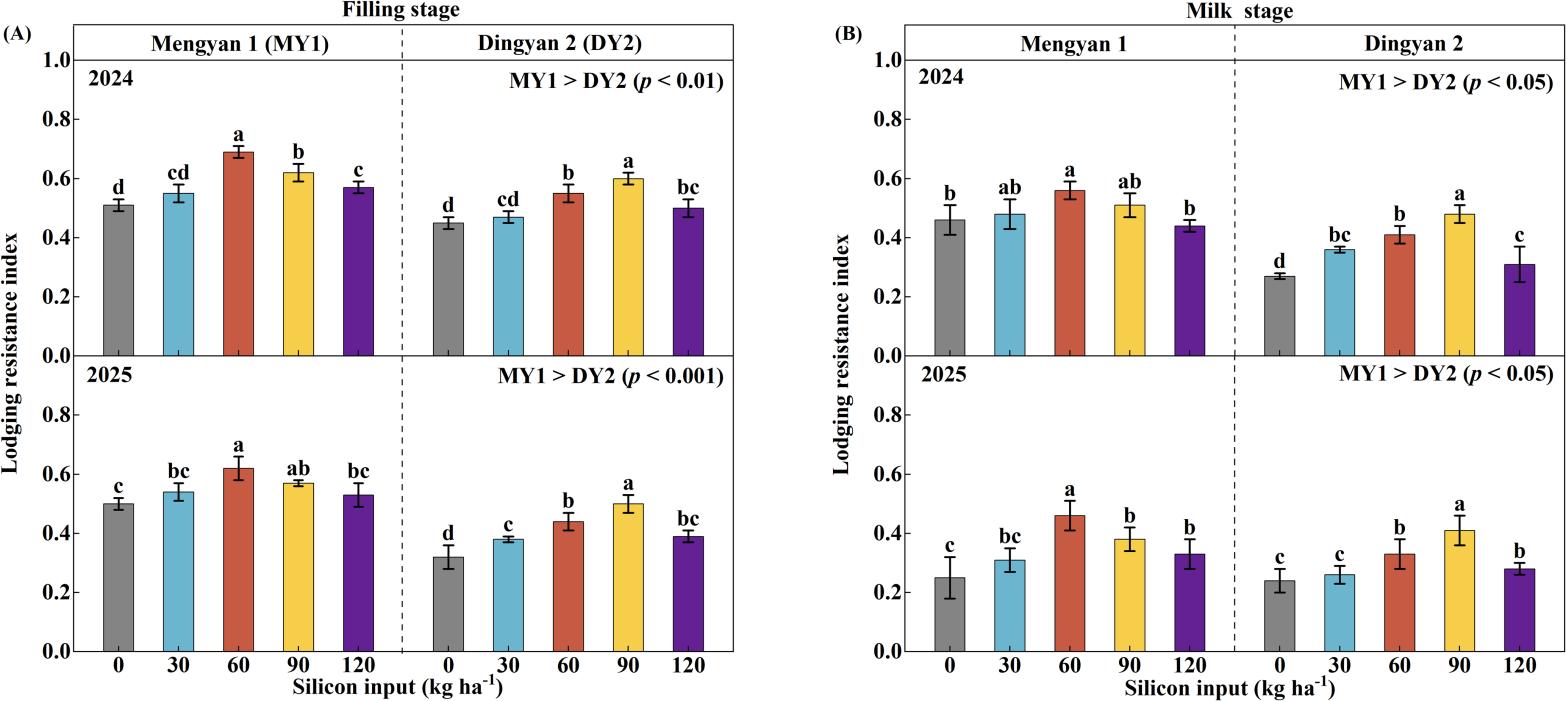
Stem lodging resistance index (LRI) of the lodging-resistant oat cultivar Mengyan 1 (MY1) and the lodging-susceptible cultivar Dingyan 2 (DY2) under different levels of silicon input. Measurements at the grain-filling stage are presented in panel **(A)**, whereas those at the milk stage are presented in panel **(B)**. Comparisons of LRI between the two varieties are presented in the upper right corner of each panel. Data are presented as means ± standard deviations (*n* = 4). Within the same year and cultivar, different lowercase letters indicate significant differences among silicon treatments according to Fisher’s least significant difference (LSD) test at *p* < 0.05.

The lodging resistance index (LRI) of oat stems at the grain-filling stage was significantly affected by year, cultivar, and their interaction (**Table 2**, *p* < 0.001). In 2024, the LRI of Mengyan 1 (MY1) and Dingyan 2 (DY2) were 0.6 and 0.5, respectively, and decreased by 6.1% and 21.0% in 2025 (**Figure 2A**). Compared with MY1, the LRI of DY2 was 12.6% and 26.5% lower in 2024 and 2025, respectively (*p* < 0.01 and *p* < 0.001). Across both experimental years, the LRI at the grain-filling and milk stages was significantly influenced by the interaction between cultivar and silicon input (**Table 2**, *p* < 0.001). The LRI of MY1 reached its maximum at a silicon input of 60 kg ha^-1^, whereas the optimal input for DY2 was 90 kg ha^-1^. On a two-year average, relative to the no-silicon control (CK), the LRI of MY1 increased by 29.7% at the grain-filling stage and by 43.7% at the milk stage, whereas that of DY2 increased by 42.9% and 74.5%, respectively (**Figure 2**, *p* < 0.05).

**Table 2:** Combined analysis of variance for the lodging resistance index and associated traits at the grain-filling and milk stages, as well as the lignin biosynthesis enzymes of the second basal internode of oat at 10, 20, 30, 40, and 50 days after jointing (DAJ).

Growth period	Traits	Source of varation						
		Year (Y)	Cultivar (cv.)	Silicon input (Si)	Y×cv.	Y×Si	cv.×Si	Y×cv.×Si
Filling stage	Lodging resistance index	***	***	***	***	ns	***	ns
	Stem breaking strength	*	***	***	**	ns	***	*
	Lignin content	***	***	***	***	ns	***	***
	K content	***	***	***	ns	**	***	ns
	Ca content	***	***	***	ns	ns	***	*
	Mg content	***	***	*	***	ns	ns	ns
	Si content	**	***	***	ns	ns	***	*
Milk stage	Lodging resistance index	***	***	***	ns	ns	***	ns
	Stem breaking strength	***	***	***	ns	*	***	ns
	Lignin content	***	***	***	ns	*	***	**
	K content	***	***	***	***	ns	***	*
	Ca content	*	***	***	ns	ns	ns	ns
	Mg content	***	***	*	***	ns	ns	ns
	Si content	***	***	***	ns	ns	**	ns
10 Days after jointing(10DAJ)	Tyrosine ammonia-lyase	***	***	***	ns	ns	**	ns
	Phenylalanine ammonia-lyase	*	***	***	**	*	ns	ns
	4-Coumarate: Coenzyme A ligase	***	***	***	ns	ns	***	***
	Cinnamyl alcohol dehydrogenase	ns	***	***	ns	**	***	*
20 Days after jointing(20DAJ)	Tyrosine ammonia-lyase	***	***	***	**	*	*	ns
	Phenylalanine ammonia-lyase	*	*	***	***	ns	***	ns
	4-Coumarate: Coenzyme A ligase	ns	ns	***	***	**	***	*
	Cinnamyl alcohol dehydrogenase	ns	***	***	ns	ns	***	*
30 Days after jointing(30DAJ)	Tyrosine ammonia-lyase	***	***	***	***	ns	**	*
	Phenylalanine ammonia-lyase	***	ns	***	***	ns	***	ns
	4-Coumarate: Coenzyme A ligase	***	***	***	*	ns	***	*
	Cinnamyl alcohol dehydrogenase	***	***	***	*	ns	***	ns
40 Days after jointing(40DAJ)	Tyrosine ammonia-lyase	ns	***	***	***	ns	***	ns
	Phenylalanine ammonia-lyase	ns	***	***	***	ns	*	*
	4-Coumarate: Coenzyme A ligase	***	***	***	***	ns	***	ns
	Cinnamyl alcohol dehydrogenase	***	***	***	ns	**	***	*
50 Days after jointing(50DAJ)	Tyrosine ammonia-lyase	***	***	***	ns	*	***	ns
	Phenylalanine ammonia-lyase	***	***	***	ns	*	ns	ns
	4-Coumarate: Coenzyme A ligase	***	***	***	***	***	***	**
	Cinnamyl alcohol dehydrogenase	ns	***	***	ns	***	***	**

*p < 0.05, **p < 0.01, ***p < 0.001, and ns indicates no significant difference.

**Figure 3:**
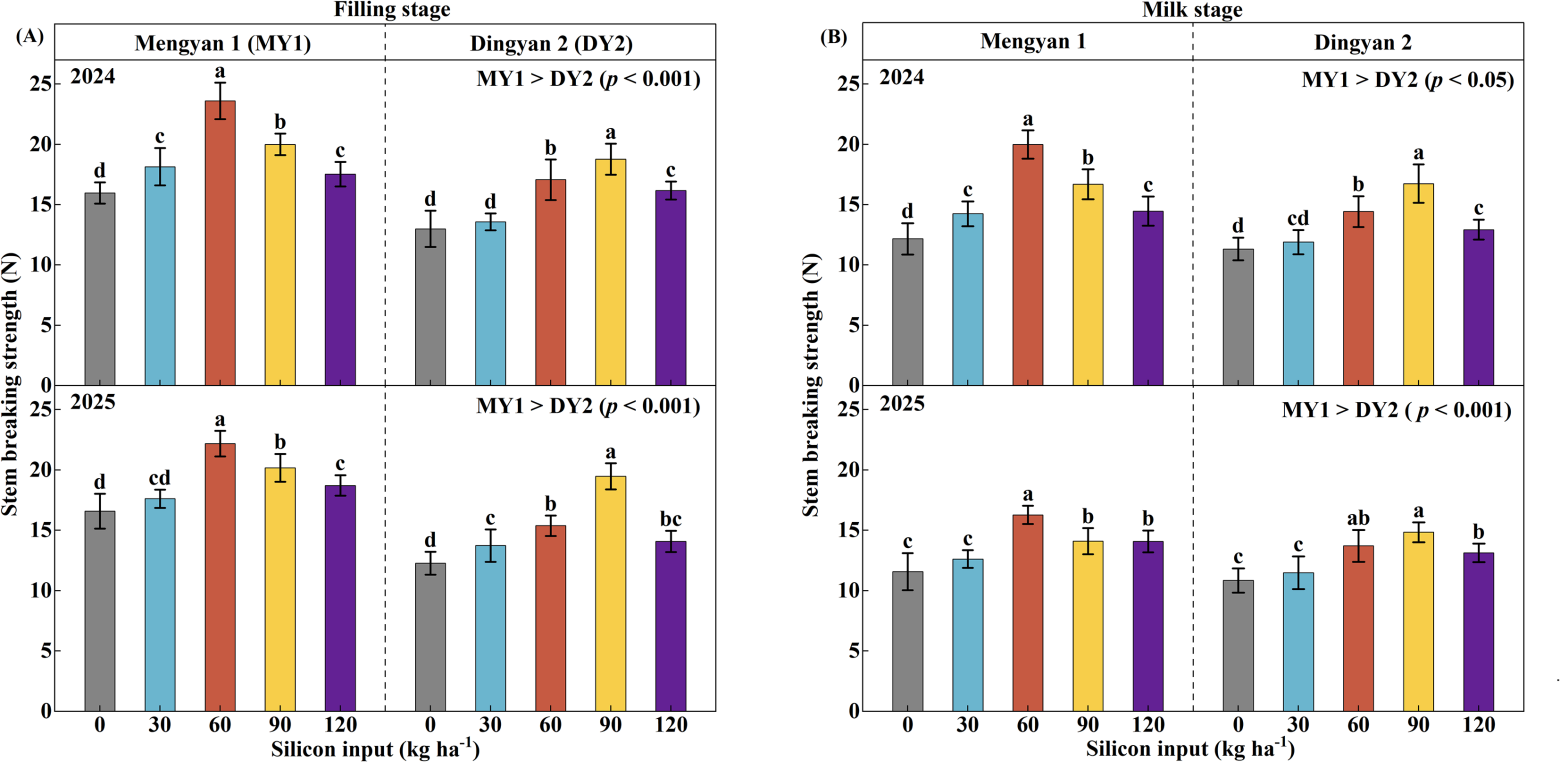
Stem breaking strength (SBS) of the lodging-resistant oat cultivar Mengyan 1 (MY1) and the lodging-susceptible cultivar Dingyan 2 (DY2) under different levels of silicon input. Measurements at the grain-filling stage are presented in panel **(A)**, whereas those at the milk stage are presented in panel **(B)**. Comparisons of SBS between the two varieties are presented in the upper right corner of each panel. Data are presented as means ± standard deviations (*n* = 4). Within the same year and cultivar, different lowercase letters indicate significant differences among silicon treatments according to Fisher’s least significant difference (LSD) test at *p* < 0.05.

Across both experimental years, the stem breaking strength (SBS) of the second basal internode was significantly influenced by cultivar, silicon input rate, and their interaction (**Table 2**, *p* < 0.001). The SBS of DY2 was 19.4% and 13.4% lower than that of MY1 at the grain-filling and milk stages, respectively (**Figure 3**, *p* < 0.001). Maximum SBS for MY1 was achieved at a silicon input rate of 60 kg ha^-1^, while for DY2 it occurred at 90 kg ha^-1^. On a two-year average, relative to the no-silicon control (CK), SBS of MY1 increased by 40.7% at the grain-filling stage and by 52.7% at the milk stage, whereas that of DY2 increased by 51.3% and 42.7%, respectively (*p* < 0.05).

### 3.2 Lignin content in the second basal internode

**Figure 4:**
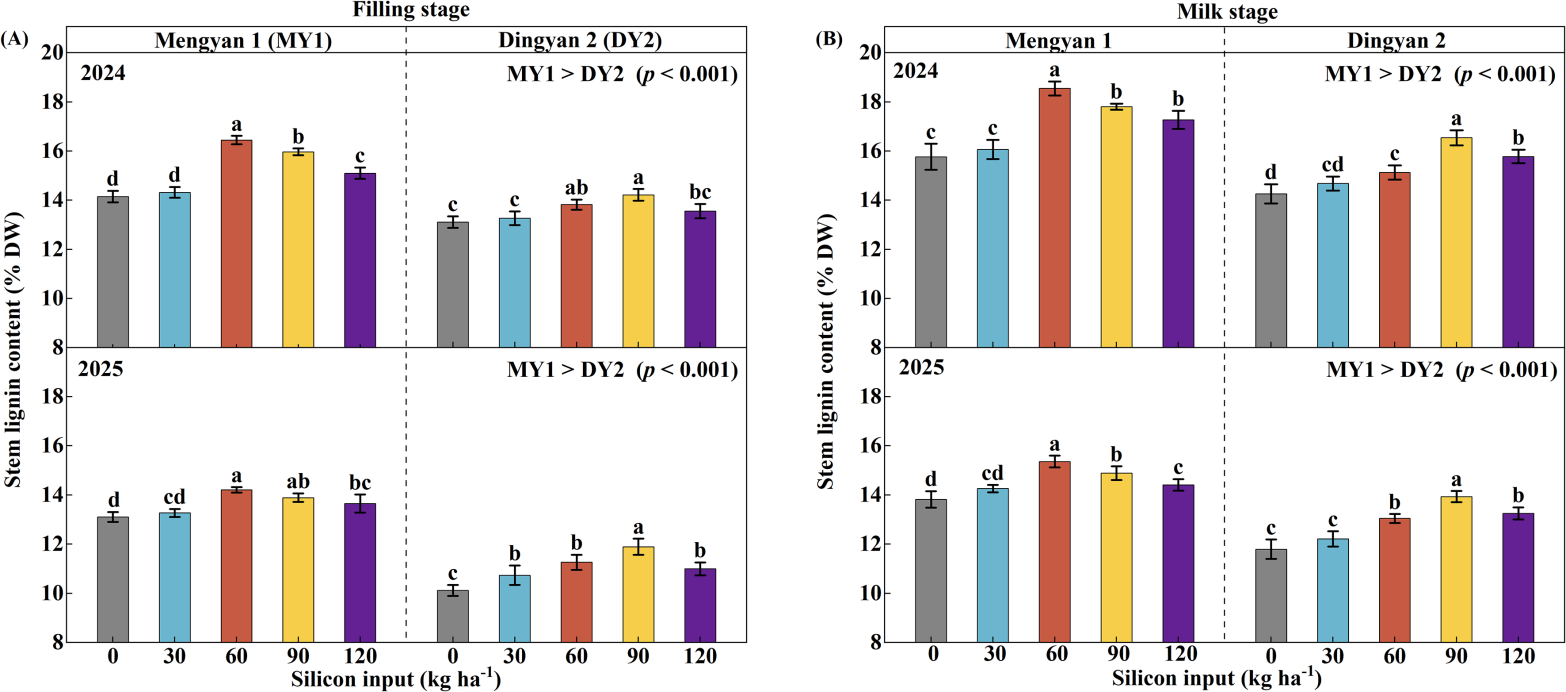
Lignin content in stems of the lodging-resistant oat cultivar Mengyan 1 (MY1) and the lodging-susceptible cultivar Dingyan 2 (DY2) under different levels of silicon input. Measurements at the grain-filling stage are presented in panel **(A)**, whereas those at the milk stage are presented in panel **(B)**. Comparisons of stem lignin content between the two varieties are presented in the upper right corner of each panel. Data are presented as means ± standard deviations (*n* = 4). Within the same year and cultivar, different lowercase letters indicate significant differences among silicon treatments according to Fisher’s least significant difference (LSD) test at *p* < 0.05.

The lignin content of the second basal internode in oat stems was significantly affected by year, cultivar, and their interaction (**Table 2**, *p* < 0.001). In 2024, the lignin contents of MY1 and DY2 were 16.1% and 14.0%, respectively, and decreased by 12.4% and 15.1% in 2025 (**Figure 4A**). Compared with MY1, the lignin content of DY2 was 12.7% and 15.3% lower in 2024 and 2025, respectively (*p* < 0.001). Across both experimental years, stem lignin content was also significantly influenced by cultivar, silicon input, and their interaction (**Table 2**, *p* < 0.001). The lignin content of MY1 reached its maximum at a silicon input of 60 kg ha^-1^, whereas the optimal input for DY2 was 90 kg ha^-1^. On a two-year average, relative to the no-silicon control (CK), lignin content of MY1 increased by 12.5% at the grain-filling stage and by 14.6% at the milk stage, whereas that of DY2 increased by 12.4% and 17.0%, respectively (**Figure 4**, *p* < 0.05).

### 3.3 Activities of lignin-biosynthetic enzymes

**Figure 5:**
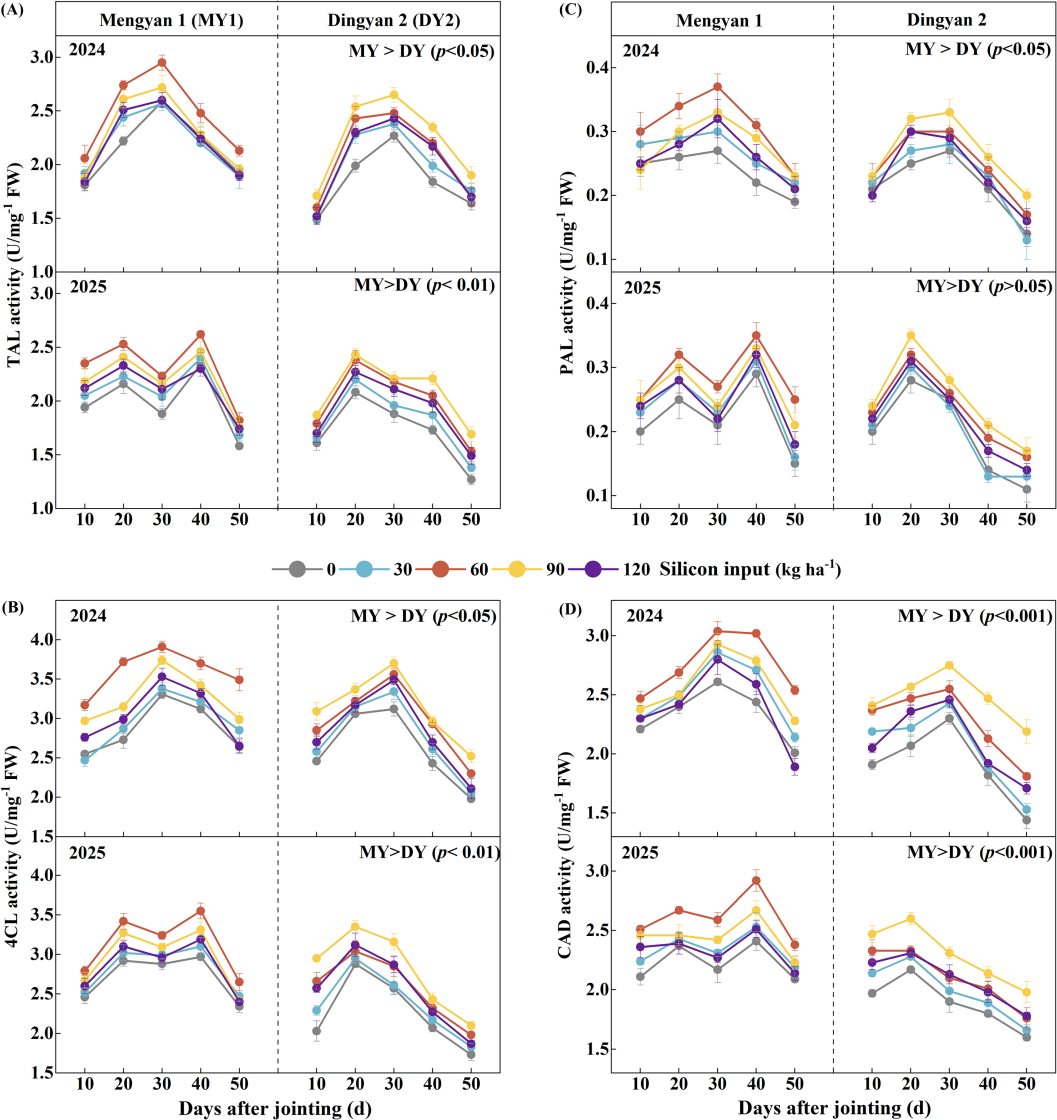
Changes in lignin-biosynthetic enzyme activities in the second basal internode of the lodging-resistant oat cultivar Mengyan 1 (MY1) and the lodging-susceptible cultivar Dingyan 2 (DY2) under different levels of silicon input at 10–50 days after jointing (DAJ). **(A, D)** represent the activities of tyrosine ammonia-lyase (TAL), 4-coumarate: CoA ligase (4CL), phenylalanine ammonia-lyase (PAL), and cinnamyl alcohol dehydrogenase (CAD), respectively. Comparisons of enzyme activities between the two cultivars are shown in the upper right of each panel. Data are presented as means ± standard deviations (*n* = 4).

The activities of lignin-biosynthetic enzymes in the second basal internode of oat stems were significantly affected by silicon input (**Table 2**, *p* < 0.001). In 2024, enzyme activities in both MY1 and DY2 followed a unimodal pattern, peaking at 30 days after jointing (DAJ). In 2025, MY1 exhibited a bimodal pattern with the highest peak at 40 DAJ, whereas DY2 remained unimodal, peaking at 20 DAJ (**Figure 5**). The activities of TAL, PAL, 4CL, and CAD in the second basal internode peaked at a silicon input of 60 kg ha^-1^ for MY1, whereas for DY2, the optimal silicon input was 90 kg ha^-1^. On a two-year average, relative to the no-silicon, the activities of TAL, PAL, 4CL, and CAD in the second basal internode increased by 15.6%, 30.6%, 20.4%, and 17.6% for MY1, and by 21.2%, 25.9%, 22.0%, and 25.9% for DY2, respectively.

### 3.4 Mineral element content

At the grain-filling stage, the K, Ca, and Si contents in the second basal internode of oat stems were significantly affected by cultivar, silicon input, and their interaction (**Table 2**, *p* < 0.001). Compared with MY1, the K, Ca, and Si contents of DY2 stems were 13.0%, 11.6%, and 6.0% lower in 2024 and 8.0%, 13.4%, and 5.6% lower in 2025, respectively (**Table 3**). The K, Ca, and Si contents in MY1 stems were highest at a silicon input of 60 kg ha^-1^, whereas the optimal rate for DY2 was 90 kg ha^-1^. On a two-year average, relative to the no-silicon, these treatments increased K, Ca, and Si contents by 25.5%, 26.1%, and 25.7% in MY1, and by 22.4%, 35.1%, and 40.3% in DY2, respectively (*p* < 0.05).

**Table 3:** K, Ca, Mg, and Si contents in oat stems at the grain-filling and milk stages under different silicon input levels.

Year	Cultivar	Silicon input (kg ha^-1^)	Filling stage				Milk stage			
			K content (g kg^-1^)	Ca content (g kg^-1^)	Mg content (g kg^-1^)	Si content (g kg^-1^)	K content (g kg^-1^)	Ca content (g kg^-1^)	Mg content (g kg^-1^)	Si content (g kg^-1^)
2024	Mengyan 1	0	13.58 ± 0.54c	0.60 ± 0.03d	0.83 ± 0.01a	6.78 ± 0.15c	10.89 ± 0.82d	0.70 ± 0.06a	0.61 ± 0.04a	7.75 ± 0.22c
		30	13.63 ± 0.30c	0.67 ± 0.01bc	0.81 ± 0.02a	7.10 ± 0.41bc	12.75 ± 0.86c	0.74 ± 0.06a	0.69 ± 0.06a	7.86 ± 0.20c
		60	17.38 ± 0.35a	0.75 ± 0.01a	0.85 ± 0.04a	8.34 ± 0.29a	16.32 ± 0.27a	0.79 ± 0.07a	0.77 ± 0.07a	9.10 ± 0.12a
		90	16.18 ± 0.16b	0.69 ± 0.02b	0.85 ± 0.05a	7.44 ± 0.37b	14.36 ± 0.59b	0.75 ± 0.08a	0.69 ± 0.06a	8.29 ± 0.11b
		120	13.89 ± 0.65c	0.66 ± 0.02c	0.80 ± 0.06a	7.71 ± 0.34b	11.74 ± 0.87cd	0.71 ± 0.05a	0.67 ± 0.06a	8.10 ± 0.30bc
	Dingyan 2	0	11.93 ± 0.52c	0.53 ± 0.01c	0.59 ± 0.03a	5.44 ± 0.25d	6.31 ± 0.58d	0.59 ± 0.05a	0.37 ± 0.08a	6.49 ± 0.42c
		30	12.89 ± 0.28bc	0.59 ± 0.02b	0.58 ± 0.03a	6.77 ± 0.30c	6.57 ± 0.62d	0.60 ± 0.06a	0.39 ± 0.02a	7.26 ± 0.40b
		60	13.04 ± 0.55b	0.61 ± 0.01b	0.61 ± 0.04a	7.35 ± 0.55bc	9.09 ± 0.61b	0.62 ± 0.07a	0.40 ± 0.05a	7.83 ± 0.36ab
		90	14.45 ± 0.12a	0.65 ± 0.01a	0.64 ± 0.05a	8.13 ± 0.19a	10.70 ± 0.62a	0.67 ± 0.04a	0.42 ± 0.05a	8.45 ± 0.39a
		120	12.63 ± 0.78bc	0.60 ± 0.02b	0.63 ± 0.06a	7.45 ± 0.24b	7.90 ± 0.30c	0.61 ± 0.03a	0.40 ± 0.04a	7.92 ± 0.15ab
2025	Mengyan 1	0	20.50 ± 0.87c	0.65 ± 0.03c	0.64 ± 0.03a	6.67 ± 0.12d	18.03 ± 0.38c	0.72 ± 0.03b	0.44 ± 0.04a	8.61 ± 0.27c
		30	22.62 ± 1.11b	0.74 ± 0.05ab	0.64 ± 0.04a	7.34 ± 0.27c	19.16 ± 1.09c	0.74 ± 0.03b	0.45 ± 0.03a	8.68 ± 0.39c
		60	25.39 ± 0.89a	0.80 ± 0.03a	0.65 ± 0.08a	8.57 ± 0.13a	22.58 ± 0.78a	0.86 ± 0.04a	0.45 ± 0.04a	9.89 ± 0.46a
		90	23.62 ± 0.67ab	0.73 ± 0.04ab	0.66 ± 0.04a	8.00 ± 0.26b	20.73 ± 0.65b	0.77 ± 0.03b	0.47 ± 0.05a	9.41 ± 0.39a
		120	23.29 ± 1.31b	0.71 ± 0.04bc	0.61 ± 0.04a	7.88 ± 0.24b	19.49 ± 0.99bc	0.71 ± 0.03b	0.46 ± 0.04a	9.26 ± 0.20ab
	Dingyan 2	0	18.77 ± 0.57c	0.54 ± 0.01c	0.56 ± 0.06ab	6.12 ± 0.14c	17.73 ± 1.35b	0.59 ± 0.04c	0.29 ± 0.01b	8.20 ± 0.40b
		30	20.82 ± 0.58b	0.59 ± 0.01c	0.48 ± 0.01b	6.97 ± 0.13b	19.02 ± 1.45ab	0.67 ± 0.05ab	0.29 ± 0.02b	8.23 ± 0.16b
		60	21.41 ± 1.40b	0.67 ± 0.04b	0.58 ± 0.09ab	7.25 ± 0.13b	20.12 ± 0.84a	0.70 ± 0.04a	0.31 ± 0.03ab	8.76 ± 0.18ab
		90	23.12 ± 0.66a	0.76 ± 0.04a	0.63 ± 0.04a	8.09 ± 0.26a	21.05 ± 0.77a	0.72 ± 0.03a	0.34 ± 0.02a	9.13 ± 0.30a
		120	22.05 ± 0.82ab	0.65 ± 0.05b	0.57 ± 0.09ab	7.86 ± 0.08a	18.10 ± 0.60b	0.61 ± 0.04bc	0.33 ± 0.04ab	8.57 ± 0.39ab

Data are presented as means ± standard deviations (*n* = 4). Within the same year and cultivar, different lowercase letters indicate significant differences among silicon input treatments according to Fisher’s least significant difference (LSD) test at *p* < 0.05.

At the milk stage, the K and Si contents in the second basal internode were significantly influenced by cultivar, silicon input, and their interaction (**Table 2**, *p* < 0.001). Compared with MY1, the K and Si contents of DY2 stems were 38.6% and 7.7% lower in 2024 and 4.0% and 6.5% lower in 2025, respectively (**Table 3**). The K and Si contents in MY1 stems were highest at a silicon input of 60 kg ha^-1^, whereas the optimal rate for DY2 was 90 kg ha^-1^. On a two-year average, relative to the no-silicon, these treatments increased K and Si contents by 34.5% and 16.1% in MY1, and by 32.1% and 19.7% in DY2, respectively (*p* < 0.05).

### 3.5 Key factors affecting lignin content

**Figure 6:**
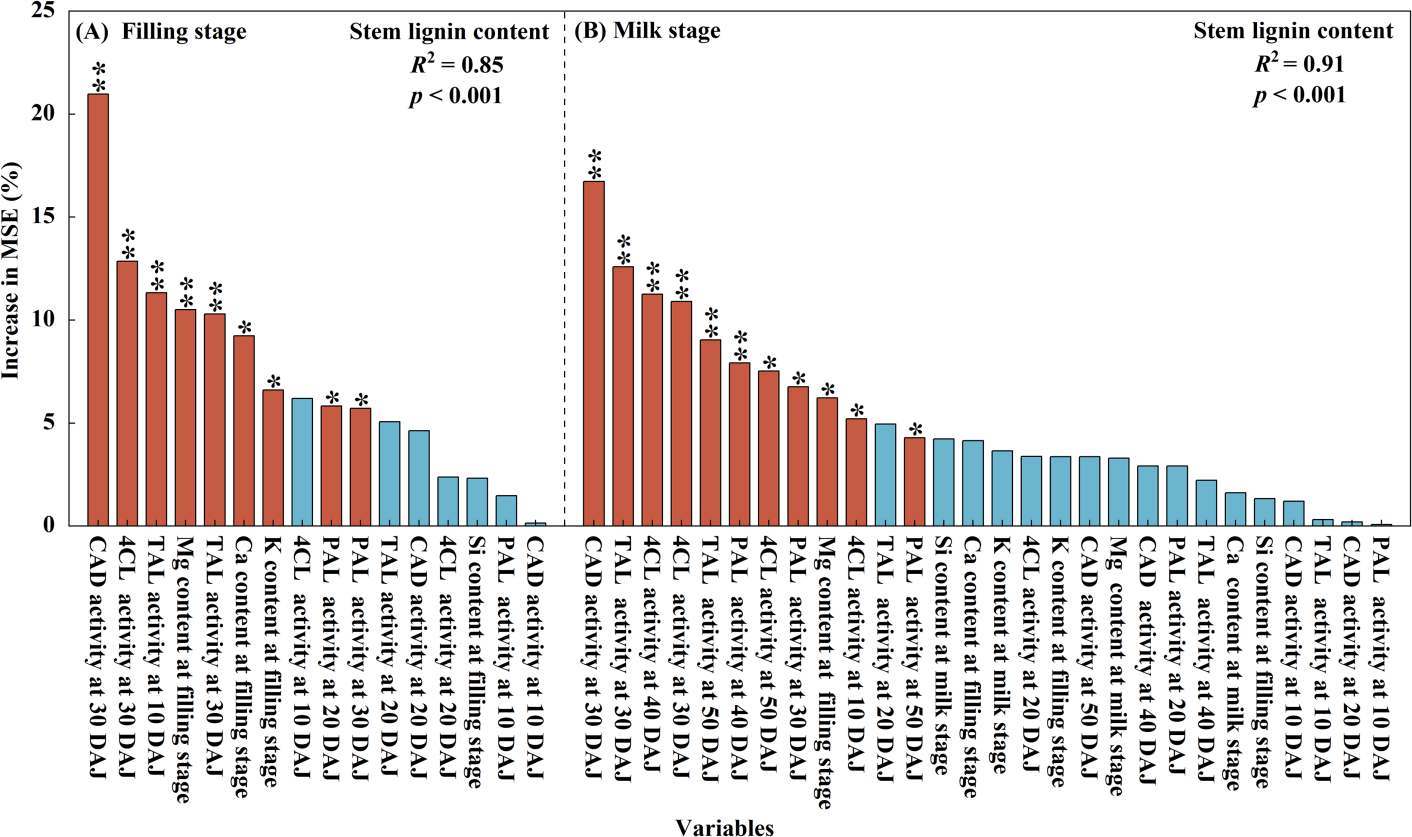
Factors affecting stem lignin content in oat at the grain-filling stage **(A)** and milk stage **(B)** as determined by the random forest model. Factors are ranked from left to right according to their relative importance. Bars are colored to indicate significance, with light red representing significant factors and light blue representing non-significant factors. * and ** indicate significance at *p* < 0.01 and *p* < 0.001, respectively.

Nine indicators were identified associated with lignin-biosynthetic at the grain-filling stage based on a random forest model (*R*^2^ = 0.85), ranked by importance as follows: CAD activity at 30 DAJ, 4CL activity at 30 DAJ, TAL activity at 10 DAJ, Mg content at grain-filling stage, TAL activity at 30 DAJ, Ca content at grain-filling stage, K content at grain-filling stage, PAL activity at 20 DAJ, and PAL activity at 30 DAJ (**Figure 6A**). In contrast, eleven key indicators were identified at the milk stage (*R*^2^ = 0.91), ranked by importance as: CAD activity at 30 DAJ, TAL activity at 30 DAJ, 4CL activity at 40 DAJ, 4CL activity at 30 DAJ, TAL activity at 50 DAJ, PAL activity at 40 DAJ, 4CL activity at 50 DAJ, PAL activity at 30 DAJ, Mg content at grain-filling stage, 4CL activity at 10 DAJ, and PAL activity at 50 DAJ (**Figure 6B**). These results indicated that lignin-biosynthetic enzyme activity at 30 days after jointing represents a critical regulatory period for lignin synthesis, with CAD activity exerting the dominant influence on stem lignin accumulation at both the grain-filling and milk stages, lignin-biosynthetic enzyme activities contributed more to lignin biosynthesis than mineral contents. The relative contributions of the indicators indicate that lignin-biosynthetic enzyme activities have a greater influence on lignin biosynthesis than mineral contents.

## 4 Discussion

### 4.1 Silicon input enhances stem lodging resistance

**Figure 7:**
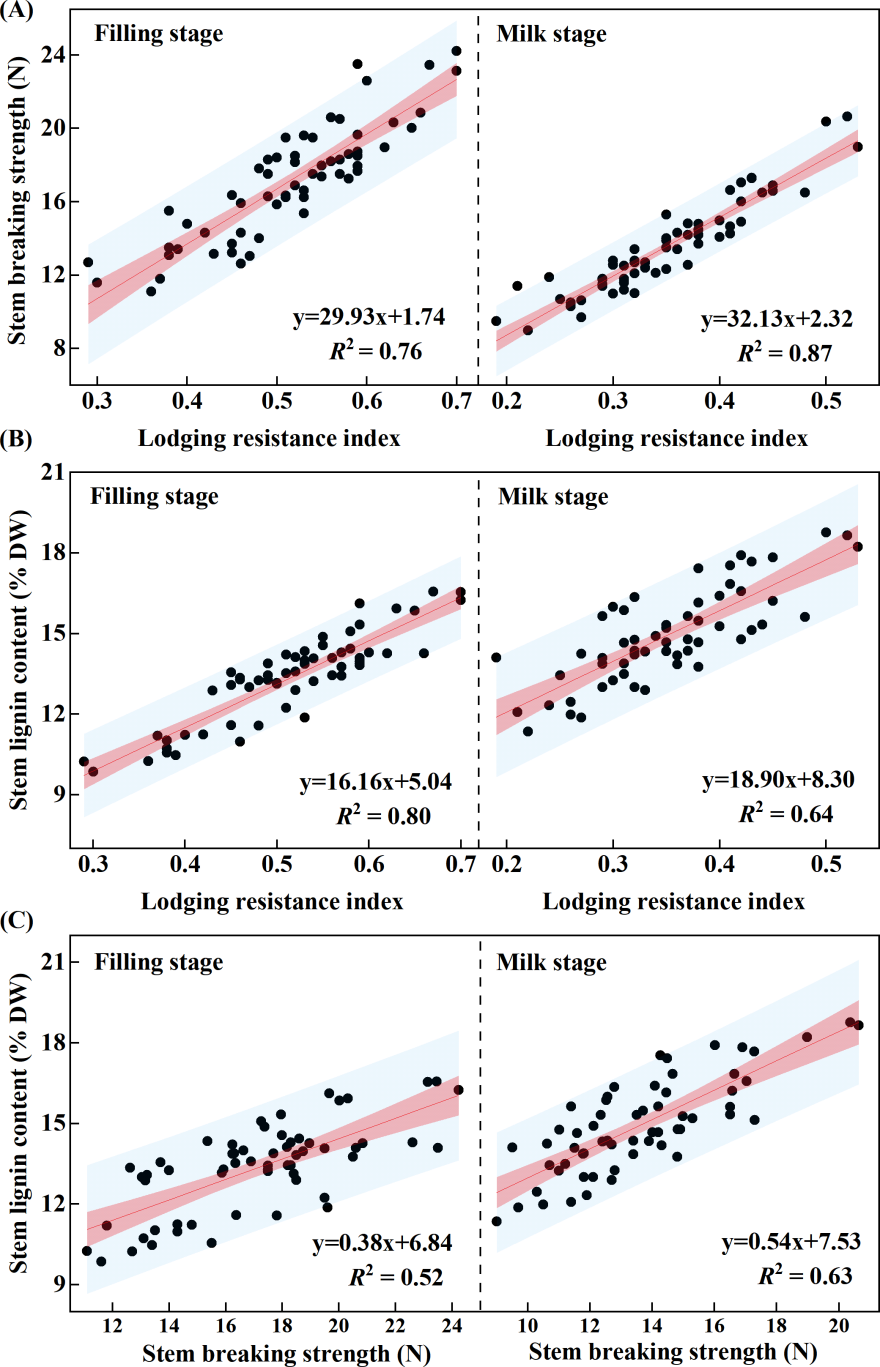
Regression analyses between stem lodging resistance index and stem mechanical strength **(A)**, stem lodging resistance index and lignin content **(B)**, and stem mechanical strength and lignin content **(C)** in oat at the grain-filling stage (left) and milk stage (right). Regression equations and *R*^2^ values are indicated at the bottom right of each panel.

The basal stem is the primary region that supports plant weight and resists external mechanical forces, and the mechanical strength of the second basal internode is considered a key determinant of lodging resistance in oat (Yu et al., 2025). Previous studies have shown that appropriate silicon input can improve the morphological structure of the basal stem and enhance stem mechanical strength, thereby increasing lodging resistance (Jiang et al., 2025; Wu et al., 2025; Yang et al., 2024). This study found that silicon input significantly affected the lodging resistance index and stem breaking strength of the second basal internode in oat (**Table 2**, *p* < 0.001), and the lodging resistance index was closely correlated with stem breaking strength during both the grain-filling stage (*R*^2^ = 0.76) and the milk stage (*R*^2^ = 0.87), consistent with previous reports (**Figure 7A**). Moreover, the optimal silicon input differed between cultivars: the lodging-resistant cultivar performed best at 60 kg·ha^-1^, whereas the lodging-susceptible cultivar performed better at 90 kg·ha^-1^ (**Figures 2**, **3**, *p* < 0.05). These differences likely reflect cultivar-specific variations in silicon uptake, transport, and utilization, which are primarily associated with the activity of silicon transporters and the expression levels of their corresponding genes (Manivannan and Ahn, 2017; Mandlik et al., 2020). Notably, regardless of silicon input level, the lodging resistance index and stem breaking strength of the lodging-resistant cultivar Mengyan 1 were consistently higher than those of the lodging-susceptible cultivar Dingyan 2, indicating that oat lodging resistance is strongly influenced by genetic characteristics (**Table 2**, *p* < 0.001). In addition, environmental factors are key determinants of crop lodging (Mohammadi et al., 2020). Year had a significant effect on the stem lodging resistance index (**Table 2**, *p* < 0.001). compared with 2024, the lodging resistance index of both cultivars decreased overall in 2025, likely due to extreme weather events, such as persistent heavy rainfall, occurring during the critical grain-filling period from July to August, which increased the incidence of lodging (**Figure 1**).

### 4.2 Silicon input increases stem lignin content and enzyme activities

Lodging in crops principally stems from insufficient stem mechanical strength, and lignin localized in the cell walls of mechanical tissues substantially increases cell-wall rigidity, thereby enhancing stem mechanical strength and lodging resistance (Liu et al., 2018b). Lignin content in the second basal internode is positively correlated with lodging resistance index and mechanical strength (Liu et al., 2024b; Luo et al., 2019). This study found that silicon input significantly increased lignin content in the second basal internode of oat stems, and lignin content was closely correlated with the lodging resistance index (**Figure 7B**, *R*^2^ = 0.80 at the grain-filling stage, *R*^2^ = 0.64 at the milk stage) and stem breaking strength (**Figure 7C**, *R*^2^ = 0.52 at the grain-filling stage, *R*^2^ = 0.63 at the milk stage).Studies in maize (*Zea mays* L.) and rice (*Oryza sativa* L.) have also shown that silicon input can increase stem lignin content and enhance stem mechanical strength, thereby reducing the risk of lodging (Shah et al., 2023; Jiang et al., 2025). Appropriate silicon input promotes integration of lignin oligomers, whereas excessive silicon may enhance oligomeric repulsion and inhibit further lignin polymerization (Djikanović et al., 2024), which aligns with the unimodal response of lignin content to increasing silicon observed in both cultivars. Moreover, lignin content in the stems of the lodging-resistant cultivar was higher than that in the lodging-susceptible cultivar, indicating that its greater lodging resistance may be attributed to lignin accumulation (Zhang et al., 2022).

Lignin biosynthesis is chiefly regulated by the activities of lignin-biosynthetic enzymes. Tyrosine ammonia-lyase, phenylalanine ammonia-lyase, 4-coumarate: CoA ligase, and cinnamyl alcohol dehydrogenase are central enzymes in the lignin-biosynthetic pathway in oat stems, and increased activities of these enzymes promote lignin accumulation, thereby enhancing stem mechanical strength and lodging resistance (Zheng et al., 2017; Wang et al., 2022). Lignin biosynthetic enzymes activities were significantly affected by silicon input (**Table 2**, *p* < 0.001), and under the optimal silicon input, both lignin content and lignin biosynthetic enzymes activities reached their highest levels in the two oat cultivars (**Figure 4** and **5**). Previous studies indicate that silicon enhances stem lodging resistance by upregulating the expression of lignin-biosynthetic genes, increasing the activities of corresponding enzymes, and thereby promoting lignin deposition to reinforce the cell-wall structure (Xue et al., 2023; Jiang et al., 2025; Shah et al., 2023). Moreover, analysis using a random forest model indicated that lignin biosynthetic enzymes activities at 30 days after jointing represent a critical regulatory period for lignin biosynthesis, and cinnamyl alcohol dehydrogenase activity plays a dominant role in stem lignin accumulation during both the grain-filling and milk stages (**Figure 6**). Previous studies indicate that the regulation of lignin biosynthesis by lignin biosynthetic enzymes is stage-dependent (Wang et al., 2022). In oat stems, internodes shift after jointing from longitudinal elongation to secondary cell wall thickening and mechanical tissue lignification, requiring increased lignin biosynthetic enzymes activity to promote lignin accumulation.

### 4.3 Silicon input promotes mineral accumulation in stems

**Figure 8:**
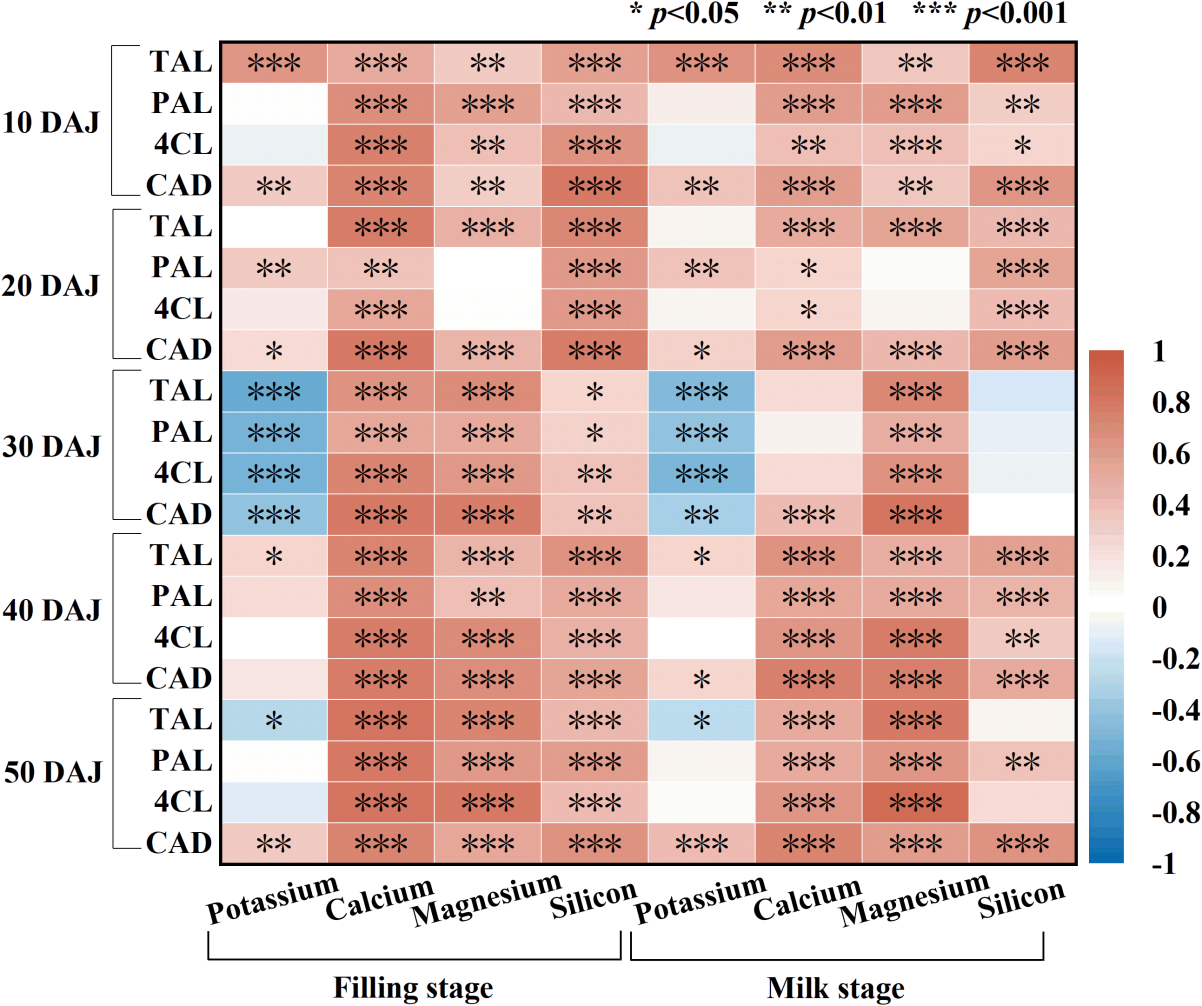
Correlation analysis between mineral contents in the second basal internode of oat stems at the grain-filling and milk stages and the activities of lignin-biosynthetic enzymes in the second basal internode at 10, 20, 30, 40, and 50 days after jointing (DAJ). TAL, tyrosine ammonia-lyase; PAL, phenylalanine ammonia-lyase; 4CL, 4-coumarate: CoA ligase; CAD, cinnamyl alcohol dehydrogenase. *p < 0.05, **p < 0.01, ***p < 0.001.

Mineral elements play a key regulatory role in crop growth and development as well as in the formation of stem mechanical strength (Liu et al., 2024a). Silicon input significantly promotes the uptake and utilization of K, Ca, and Si in crops (Kupdhoni et al., 2025), which is consistent with the results of the present study. Minerals in stems contribute to lodging resistance through two main pathways. First, they activate genes involved in lignin biosynthesis and enhance the activities of lignin-biosynthetic enzymes, thereby promoting lignin accumulation. Second, they are deposited in cell walls or intercellular spaces through processes such as calcification and silicification, which directly strengthen stem mechanical properties (Huang et al., 2023; Ma et al., 2025; Wu et al., 2023). This study showed that stem Mg content responded weakly to silicon input, with a significant effect only in Dingyan 2 in 2025 (**Table 2**, *p<* 0.05), possibly because silicon promotes Mg uptake only under Mg deficiency (Buchelt et al., 2020). However, the random forest model identified Mg as a key indicator, suggesting that its role in lignin synthesis may not depend on accumulation, but may indirectly promote lignin synthesis in a catalytic manner. Across the two-year trial, stem K content in both cultivars was significantly higher in 2025 than in 2024, which may be attributable to intensified lodging in 2025, leading to the redistribution and accumulation of K due to its high mobility (Mostofa et al., 2022). Excessive K can inhibit the activity of lignin-biosynthetic enzymes through ion competition or signal regulation, thereby reducing lignin accumulation (Wu et al., 2023), which is consistent with the significant negative correlation observed in this study between stem K content and lignin-biosynthetic enzyme activity at 30 days after jointing (**Figure 8**). Overall, silicon input promoted mineral accumulation in oat stems, and Ca, Mg, and Si were significantly and positively correlated with the activities of lignin-biosynthetic enzymes, indicating that mineral elements contribute, at least in part, to the activation of these enzymes and thereby enhance lignin biosynthesis. However, the specific physiological mechanisms by which individual minerals regulate lignin biosynthesis and lodging resistance remain to be further systematically elucidated.

## 5 Conclusions

Silicon input significantly enhanced stem mechanical strength and lodging resistance in oat, primarily by promoting lignin accumulation in the second basal internode. Silicon input increased lignin biosynthetic enzymes activities and stem mineral content. Thirty days after jointing represents a critical period for the regulation of lignin biosynthesis, with cinnamyl alcohol dehydrogenase activity contributing most to lignin accumulation. Compared with enzyme activity, minerals had a smaller contribution to lignin biosynthesis, with Mg content during the grain-filling stage identified as a key mineral factor by the random forest model. Silicon inputs of 60 and 90 kg ha^-1^ are recommended for lodging resistant and susceptible oat cultivars respectively, and it enhances lodging resistance by the promotion of lignin accumulation through upregulating enzyme activities and increasing mineral content in the stems.

## Data Availability

The raw data supporting the conclusions of this article will be made available by the authors, without undue reservation.
